# Acceptability and Feasibility of Self-Collecting Biological Specimens for HIV, Sexually Transmitted Infection, and Adherence Testing Among High-Risk Populations (Project Caboodle!): Protocol for an Exploratory Mixed-Methods Study

**DOI:** 10.2196/13647

**Published:** 2019-05-02

**Authors:** Akshay Sharma, Rob Stephenson, Gregory Sallabank, Leland Merrill, Stephen Sullivan, Monica Gandhi

**Affiliations:** 1 Center for Sexuality and Health Disparities University of Michigan School of Nursing Ann Arbor, MI United States; 2 Department of Health Behavior and Biological Sciences University of Michigan School of Nursing Ann Arbor, MI United States; 3 Department of Systems, Population and Leadership University of Michigan School of Nursing Ann Arbor, MI United States; 4 Division of HIV, Infectious Disease, and Global Medicine Department of Medicine University of California, San Francisco San Francisco, CA United States

**Keywords:** HIV infections, sexually transmitted diseases, pre-exposure prophylaxis, social networking, sexual minorities

## Abstract

**Background:**

Men who have sex with men (MSM) in the United States experience a disproportionate burden of HIV and bacterial sexually transmitted infections (STIs), such as gonorrhea and chlamydia. Screening levels among MSM remain inadequate owing to barriers to testing such as stigma, privacy and confidentiality concerns, transportation issues, insufficient clinic time, and limited access to health care. Self-collection of specimens at home and their return by mail for HIV and bacterial STI testing, as well as pre-exposure prophylaxis (PrEP) adherence monitoring, could be a resource-efficient option that might mitigate some of these barriers.

**Objective:**

Project Caboodle! is a mixed-methods study that explores the acceptability and feasibility of self-collecting and returning a bundle of 5 different specimens for HIV and bacterial STI testing, as well as PrEP adherence monitoring, among sexually active HIV-negative or unknown status MSM in the United States aged 18 to 34 years.

**Methods:**

Participants will be recruited using age, race, and ethnicity varied advertising on social networking websites and mobile gay dating apps. In Phase 1, we will send 100 participants a box containing materials for self-collecting and potentially returning a finger-stick blood sample (for HIV testing), pharyngeal swab, rectal swab, and urine specimen (for gonorrhea and chlamydia testing), and hair sample (to assess adequacy for potential PrEP adherence monitoring). Specimen return will not be incentivized, and participants can choose to mail back all, some, or none of the specimens. Test results will be delivered back to participants by trained counselors over the phone. In Phase 2, we will conduct individual in-depth interviews using a video-based teleconferencing software (VSee) with 32 participants from Phase 1 (half who returned all specimens and half who returned some or no specimens) to examine attitudes toward and barriers to completing various study activities.

**Results:**

Project Caboodle! was funded in May 2018, and participant recruitment began in March 2019. The processes of designing a study logo, creating advertisements, programming Web-based surveys, and finalizing step-by-step written instructions accompanied by color images for specimen self-collection have been completed. The boxes containing 5 self-collection kits affixed with unique identification stickers are being assembled, and shipping procedures (for mailing out boxes to participants and for specimen return by participants using prepaid shipping envelopes) and payment procedures for completing the surveys and in-depth interviews are being finalized.

**Conclusions:**

Self-collection of biological specimens at home and their return by mail for HIV and bacterial STI testing, as well as PrEP adherence monitoring, might offer a practical and convenient solution to improve comprehensive prevention efforts for high-risk MSM. The potentially reduced time, expense, and travel associated with this approach could facilitate a wider implementation of screening algorithms and remote monitoring strategies.

**International Registered Report Identifier (IRRID):**

PRR1-10.2196/13647

## Introduction

### Background

Despite representing a small fraction of the US population [1,2], men who have sex with men (MSM) account for 58.95% (648,500/1.1 million) of all people living with HIV [3] and 66.79% (26,570/39,782) of all new HIV diagnoses annually [4]. According to the Centers for Disease Control and Prevention (CDC), HIV diagnoses among MSM increased from 25,155 in 2005 to 26,612 in 2014 [5]. The rate of incident diagnoses in this risk group is more than 44 times that of other men [3], and the rate of prevalent diagnoses is more than 57 times greater [6]. MSM in the United States also face a high burden of bacterial sexually transmitted infections (STIs), such as gonorrhea and chlamydia. Prevalence proportions for pharyngeal, rectal, and urethral gonorrhea among MSM are estimated to be 7.90% (1,144/14,484), 10.24% (1,136/11,092), and 11.14% (2,056/18,460), respectively, and those for pharyngeal, rectal, and urethral chlamydia are estimated to be 2.86% (199/6,961), 14.14% (1,427/10,091), and 8.35% (1,495/17,898), respectively [7]. These data are concerning because bacterial STIs acting through biological mechanisms, in addition to behaviors associated with their acquisition and transmission, are well-established risk factors for HIV [8-12].

The CDC currently recommends at least annual screening for HIV, gonorrhea, and chlamydia for all sexually active MSM [13,14]. More frequent screening for HIV (eg, at 3- to 6-month intervals) could be offered to those at increased risk based on an assessment of their individual risk factors, local HIV epidemiology, and local testing policies [13,15]. Testing is the first step in offering pre-exposure prophylaxis (PrEP) for HIV prevention to those who test negative or initiating treatment for HIV and other STIs among those who test positive. However, screening levels among MSM remain inadequate [16,17] owing to barriers to testing such as stigma, privacy and confidentiality concerns, transportation issues, insufficient clinic time, and limited access to health care [18-30]. In 2014, 71.14% (5,864/8,243) of MSM participating in the National HIV Behavioral Surveillance reported testing for HIV in the past year [17], and 46.98% (8,984/19,124) reported testing for gonorrhea or chlamydia in the past year [31]. Novel strategies are needed to increase current rates of HIV and other STI screening among MSM. Self-collection of specimens at home and their return by mail for laboratory testing could be a resource-efficient option [32] and has the potential to reduce both personal and logistical barriers to regular testing [33].

Oral PrEP with tenofovir (TFV) disoproxil fumarate and emtricitabine (FTC) significantly reduces the risk of HIV among MSM but relies on adequate adherence for effectiveness [34-38]. Measuring biomarkers in biological specimens for assessing PrEP adherence played a key role in the interpretation of landmark placebo-controlled randomized clinical trials [39-41]. The delivery of proven biomedical interventions needs to be accompanied by complementary strategies for measuring and increasing adherence to optimize their effectiveness [42-46]. Although recent demonstration projects have reported high levels of PrEP use and adherence in the context of known efficacy [38,47], adherence biomarkers need to be incorporated into PrEP implementation and roll-out programs to assess its effectiveness in real-world settings and to tailor adherence interventions [48,49]. Hair samples can be used for PrEP adherence measurement [48-58], both in daily and in event-driven PrEP users. As distance along the hair shaft serves as a marker of time, the segmental analysis of hair samples allows for an objective assessment of event-driven PrEP adherence at various time points over previous months [59,60]. Self-collection at home and return by mail of this nonbiohazardous, easy-to-ship specimen that is stable at room temperature might facilitate remote PrEP adherence monitoring and thereby allow for the appropriate identification of MSM facing adherence difficulties for intervention.

Numerous studies on biological specimen self-collection have been conducted in clinical settings, wherein patients immediately return their samples to clinic staff for subsequent testing in a laboratory. For example, self-collected nasal swabs have been used to diagnose respiratory tract infections [61-63], self-collected vaginal swabs have been used for bacterial STIs and cervical cancer screening among women [64-71], and self-collected genital specimens have been used for human papillomavirus screening among men [72-74]. This approach prioritizes patient comfort and facilitates clinic flow, but it cannot reach individuals who do not come to the clinic for testing. Recently, there has been an increase in research focusing on home specimen self-collection for HIV and other STI testing among diverse populations, including sexual minorities [75-87]. Several of these studies involved either cross-sectional surveys or focus group discussions to assess the acceptability of this approach but few studies have also examined feasibility, that is, whether MSM would actually return individual specimens and whether these specimens would be adequate for laboratory testing. To our knowledge, no study has evaluated the acceptability and feasibility of self-collecting at home and returning by mail a bundle of different types of biological specimens that can be used for HIV and bacterial STI screening (eg, a finger-stick blood sample, pharyngeal swab, rectal swab, and urine specimen) and for potential PrEP adherence monitoring (eg, a hair sample) among young sexually active MSM.

### Objectives

Combining the self-collection of biological specimens from different anatomical sites for HIV and bacterial STI testing along with the self-collection of a hair sample, a specimen that has been shown to have utility in PrEP adherence measurement [48-58], could hold promise as a remote monitoring strategy for individuals at risk. Gathering and evaluating data on the specimens that MSM are willing to self-collect at home, the ones they actually mail back, and the adequacy of returned specimens for laboratory testing are critical to developing interventions to help increase HIV and other STI screening rates, as well as adherence to PrEP in this high-risk group. The purpose of this paper is to describe the protocol for an innovative mixed-methods study seeking to evaluate the acceptability and feasibility of biological specimen self-collection and return from a diverse sample of young sexually active HIV-negative or unknown status MSM in the United States. The procedures described below have been reviewed and approved by the Institutional Review Board at the University of Michigan in Ann Arbor (HUM00153673) and have been deemed to pose no more than minimal risk to study participants.

## Methods

### Study Overview

Project Caboodle! is an exploratory 2-year study to obtain both quantitative and qualitative data regarding the acceptability and feasibility of biological specimen self-collection and return from sexually active HIV-negative or unknown status MSM aged 18 to 34 years residing in the United States or dependent areas. In Phase 1, we will send 100 participants a box containing instructions and materials for self-collecting and potentially returning a finger-stick blood sample (for HIV testing), pharyngeal swab, rectal swab, and urine specimen (for gonorrhea and chlamydia testing), and hair sample (to assess adequacy for potential PrEP adherence monitoring in the future). Specimen return is not incentivized, and participants can choose to mail back all, some, or none of these 5 specimens. In Phase 2, we will conduct individual in-depth interviews using a video-based teleconferencing software (VSee) with 32 participants from Phase 1 to examine attitudes toward and barriers to self-collecting and returning each type of specimen.

Guided by the theoretical constructs of the Information-Motivation-Behavioral skills (IMB) model [88,89], the specific aims of our study are as follows:

1. *Aim 1:* Explore the acceptability and feasibility of self-collecting at home and returning by mail all, some, or none of the following biological specimens: (1) finger-stick blood sample, (2) pharyngeal swab, (3) rectal swab, (4) urine specimen, and (5) hair sample, among 100 sexually active HIV-negative or unknown status US MSM (aged 18 to 34 years, 50 black and 50 white including those of Hispanic ethnicity) recruited through social media platforms.
2. *Aim 2:* Collect qualitative data via individual in-depth interviews conducted using VSee from 2 subsamples of 16 MSM each, including participants who (1) returned all 5 specimens and (2) returned some or no specimens to examine attitudes toward and barriers to completing various study activities.

### Theoretical Approach

Our study is grounded in the IMB model of HIV prevention [88] that describes pivotal constructs pertaining to health self-management [89]. Theoretical constructs of the IMB model will be applied to explore the acceptability and feasibility of self-collecting and potentially returning the 5 different types of specimens by MSM. *Information* will be assessed through a baseline survey in Phase 1, measuring pre-existing awareness of commercially available self-collection kits using questions adapted from our past research on rapid home HIV testing using oral fluid and finger-stick blood samples [90,91]. The survey will also assess participants’ knowledge of transmission and prevention of HIV and other STIs using the Sexually Transmitted Disease Knowledge Questionnaire (STD-KQ) [92]. *Motivation* will be examined through questions in the baseline survey on participants’ theoretical willingness to self-collect and return each type of specimen, expanding upon our previous study focusing exclusively on dried blood spot specimens [86] and by calculating the proportions of each type of specimen actually returned for laboratory testing. Depending upon their inclination, participants can choose to mail back all, some, or none of the 5 specimens. *Behavioral skills* will be measured by assessing the adequacy of returned specimens for current and future laboratory testing, thereby evaluating the ability of participants to self-collect and return useful specimens without supervision. Theoretical constructs of the IMB model will also be explored through qualitative in-depth interviews in Phase 2, which will focus on familiarity with the concept of biological specimen self-collection (information) and attitudes toward self-collecting and returning each type of specimen (motivation), as well as personal and logistical barriers encountered while performing study activities (behavioral skills).

### Participant Recruitment

Project Caboodle! will recruit and enroll 100 young (aged 18 to 34 years), sexually active HIV-negative or unknown status MSM residing in the United States. Participants will be recruited online using age-appropriate, racially and ethnically diverse advertising on social networking websites (eg, Facebook and Instagram) and mobile gay dating apps (eg, Grindr and Scruff). We will aim to ensure that our sample includes equal numbers of black and white MSM including those of Hispanic ethnicity. Our advertisements will include images of men hugging, kissing, or holding hands, the study logo, and call-to-action text. These will appear as posts on social networking websites and will be targeted to profiles reflecting gay *interests*, that is, topics users have accessed (eg, same-sex marriage), and pages they have *liked* (eg, pride events).

Individuals who click on our advertisements will be directed to our study’s landing page (programmed in Qualtrics, a secure Web-based platform approved by the University of Michigan) that will provide a brief overview of the study protocol, in addition to basic information on the burden of HIV and bacterial STIs among MSM in the United States. Those who are not interested can exit by closing the landing page in their browser. Interested individuals can click a button to continue, which will direct them to a comprehensive informed consent document. This document will provide information regarding our study’s purpose, contents of the eligibility screener, the baseline survey, and the posttest survey, study procedures in Phases 1 and 2, potential risks of participation, and the right to refuse participation or withdraw at any time. Individuals will be asked to provide consent to (1) be screened for eligibility, (2) be asked for their contact information, (3) participate in the biological specimen self-collection activities (ie, Phase 1) if they meet the eligibility criteria, and (4) be potentially asked to participate in an in-depth interview (ie, Phase 2).

Individuals who consent will be asked to complete an 8-question eligibility screener, programmed in Qualtrics. The eligibility criteria include the following: (1) assigned male sex at birth, (2) currently identify as male, (3) aged 18 to 34 years (owing to the high burden of HIV and bacterial STIs among MSM in this age group [4,5]), (4) currently reside in the United States or dependent areas, (5) are a legal adult in their state of residence, (6) report HIV-negative or unknown status, (7) had ≥2 male sex partners in the past 3 months, and (8) are willing to receive a box containing instructions and materials for specimen self-collection at their preferred mailing address. Eligible MSM will then be asked to register by providing their full name, email address, and mobile phone number. They will also be asked to indicate whether they prefer being contacted by email, phone call, or text to remind them to complete Phase 1 surveys (baseline and posttest) and to be potentially invited to participate in Phase 2 in-depth interviews. Finally, they will be asked to provide a mailing address where they would be willing to receive a box containing instructions and materials for self-collecting and potentially returning different types of biological specimens.

Potential participants’ identities will be verified using 3 steps. First, the Internet Protocol (IP) address of the device that was used to complete the eligibility screener will be recorded within Qualtrics and checked by the study staff to verify that (1) the IP address location is within the United States and (ii) there are no duplicate entries from the same IP address. Second, each potential participant will be asked to reply to an email sent to their preferred email address to confirm its accuracy and functionality. Third, the email address, mobile phone number, and mailing address provided will be validated using Spokeo, a Web-based search platform that aggregates publicly available social media and archival data. Spokeo will be used to further authenticate that the email address and mobile phone number provided by a potential participant correctly links to their provided full name and key demographic eligibility criteria (eg, cisgender male identity). To continue as a participant in the study, one needs to (1) have their IP address located in the United States, (2) reply to the confirmation message sent to their provided email address, (3) have at least 1 aspect of their provided contact information (email address, mobile phone number, and mailing address) be linked to their provided full name or key demographic eligibility criteria. Individuals whose identities cannot be verified will be sent an email informing them that they cannot continue in the study and thanking them for their interest. Such email notifications will take place on an ongoing basis immediately after the verification process is complete.

**Figure 1:**
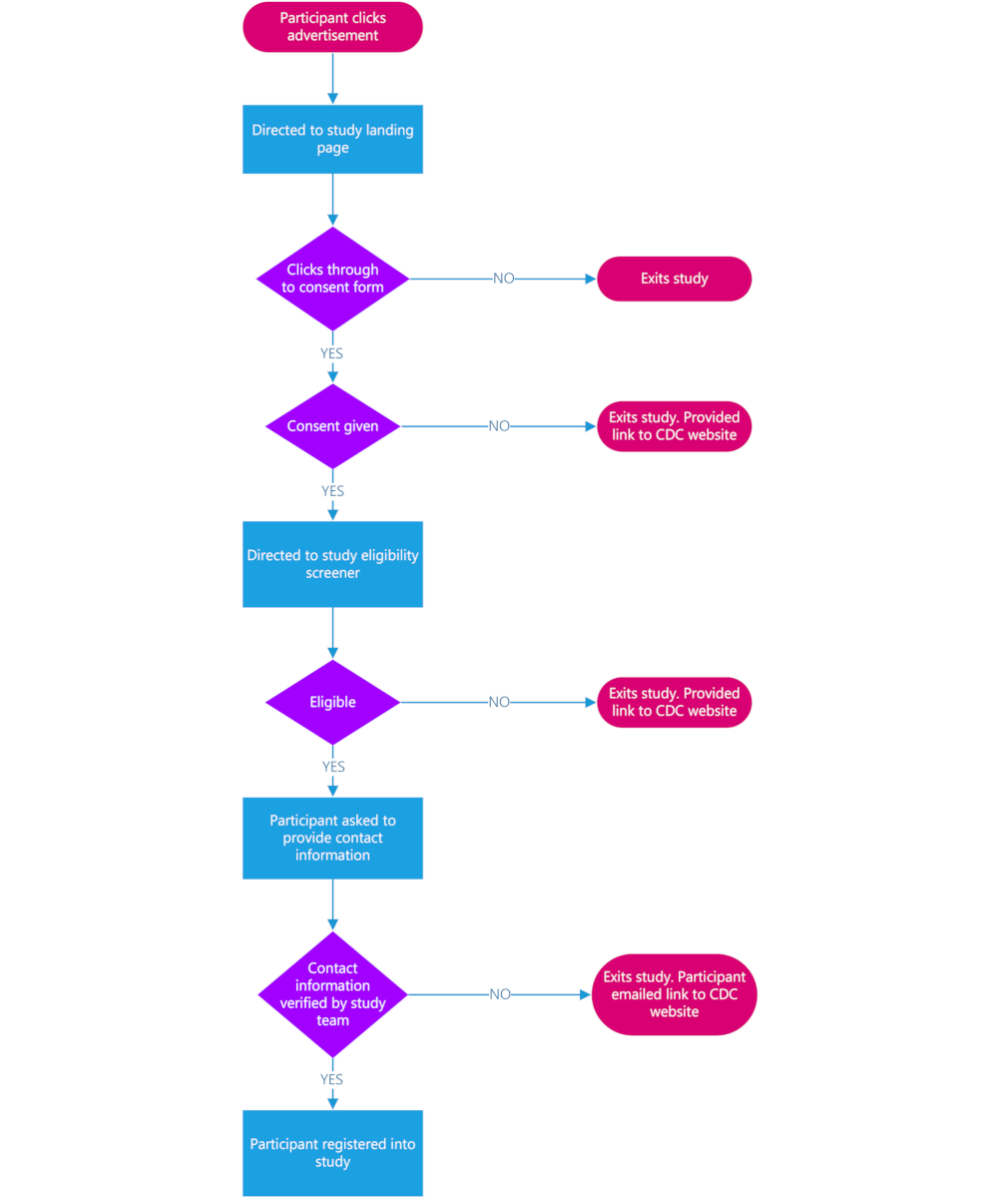
Participant recruitment process for Project Caboodle!. CDC: Centers for Disease Control and Prevention.

Individuals who do not consent, do not meet the eligibility criteria, or do not provide valid contact information will be excluded from participating any further and will be directed to the CDC HIV Risk Reduction Tool website containing links to sexual health information, PrEP, and other HIV and STI prevention resources [93]. Those who consent, who meet the eligibility criteria, and whose identities have been verified will be registered as study participants (see Figure 1).

### Phase 1 Procedures

Registered participants will be sent an email containing a link to the baseline survey, programmed in Qualtrics. The survey will include questions pertaining to the following domains— *demographic characteristics:* data on age, race and ethnicity, educational level, sexual orientation, employment status, history of incarceration, history of homelessness, health insurance coverage, and access to health care will be collected [94]; *HIV testing history:* participants will be asked about whether they have ever been tested for HIV, the time frame of their most recent test, the location of their most recent test, and their annual frequency of testing or their reasons for never testing [81,86,94]; *home-based HIV testing:* data will be collected on whether participants have ever used a commercially available self-collection kit (eg, Home Access HIV-1 Test System and OraQuick In Home HIV Test) and the type of test kit they have used or their reasons for never using a self-collection kit; *PrEP knowledge, use, and intentions:* questions will be adapted from recent studies of PrEP attitudes among MSM [95-97] to collect data on whether participants have heard about PrEP, are currently using PrEP, their previous use of PrEP, reasons for never using or discontinuing PrEP, access to PrEP, and intentions for future use of PrEP; *bacterial STI testing history:* participants will be asked about whether they have ever been tested for bacterial STIs, the time frame of their most recent tests, the location of their most recent tests, different anatomical sites at which they have been tested, whether they have ever been diagnosed with any STIs in the past, and if so, whether they received treatment or their reasons for never testing [98,99]; *sexual behavior:* data will be collected on relationship status, the number of sex partners with whom participants have had condom-less anal or oral sex in the past 3 months, and whether any of those partners were known to be positive for HIV or other STIs [100,101]; *knowledge of transmission and prevention of HIV and other STIs:* the 27-item STD-KQ will be used to assess participants’ knowledge about different STIs [92]; *perceived risk of contracting HIV and other STIs:* participants will be asked to indicate how concerned they are about contracting HIV and other STIs on a 10-point scale ranging from not concerned at all to very concerned [102]; *experiences with medical care:* the Group-Based Medical Mistrust Scale will be used to assess participants’ previous experiences of racial discrimination and feelings of discomfort and suspicion they might have toward health care personnel and medical treatments [103]; *theoretical willingness and perceived ability to self-collect and return biological specimens:* Likert-item questions will be used to assess participants’ intentions to self-collect and return each of the 5 different types of biological specimens similar to previous studies [81,90], as well as their confidence to complete these activities using simple step-by-step written instructions accompanied by color images; *substance use and psychological distress:* participants will be asked about their alcohol, tobacco, and other drug use in the past 6 months and to self-report depressive symptomology on the revised Center for Epidemiologic Studies Depression Scale [104]. The baseline survey will take approximately 30 min to complete, and participants will receive an incentive of US $40 in the form of an Amazon gift card.

After participants have been sent the original email containing a link to the baseline survey, they will receive up to 3 reminders at weekly intervals using their preferred communication method indicated during registration (email, phone call, or text). The final reminder will advise participants that noncompletion of the survey within the next 7 days will suggest that they are no longer interested in continuing, unless they contact the study staff and ask for an extension. They will be also be advised that they will no longer qualify to receive a specimen self-collection box in the mail after that time has lapsed. Participants who do not complete the baseline survey within 7 days of receiving the final reminder (ie, by the end of 4 weeks after the original email has been sent) will be thanked for their interest in the study and emailed information about the CDC HIV Risk Reduction Tool website.

Participants who complete the baseline survey will be shipped a box containing instructions and materials for self-collecting and potentially returning 5 different types of biological specimens (ie, a finger-stick blood sample, pharyngeal swab, rectal swab, urine specimen, and hair sample). Each box and its components will be affixed with a unique identification sticker to enable specimen identification upon return. Boxes will be shipped from the University of Michigan School of Nursing (UMSN) in Ann Arbor using United Parcel Service (UPS) in plain unmarked packaging, and their delivery will be tracked by the study staff on an ongoing basis. Contents of the box will include the following:

#### General Instructions

Participants will be provided with a brief overview of the study procedures and a description of the 5 specimen self-collection kits contained in the box. They will be informed that they can choose to self-collect and return all, some, or none of the specimens depending on their comfort levels. They will also be informed that the box includes 2 prepaid shipping envelopes affixed with FedEx labels—#1 for biological specimens to be returned to the Emory University Clinical Virology Research Laboratory in Atlanta for HIV, gonorrhea, and chlamydia testing (ie, a finger-stick blood sample, pharyngeal swab, rectal swab, and urine specimen) and #2 for a hair sample to be returned to the Hair Analytical Laboratory (HAL) at the University of California, San Francisco (UCSF).

#### Specific Instructions

Each specimen self-collection kit will contain simple step-by-step written instructions accompanied by color images provided by the laboratories.

1. Finger-stick blood sample self-collection kit (for HIV testing): Participants will be instructed to wash their hands with soap and warm water, clean their middle or ring finger from their nondominant hand using the alcohol wipe, stimulate blood flow by shaking their hand below the waist for 15 seconds, prick their chosen finger using the safety lancet, wipe away the first drop of blood using the sterile gauze, collect blood using the capillary into the transport tube marked with a fill line, secure the transport tube’s cap, apply the bandage to their finger, gently mix the blood with the anticoagulant in the transport tube by flipping it upside down, and finally place the tube in the provided biohazard bag.
2. Pharyngeal swab self-collection kit (for gonorrhea and chlamydia testing): Participants will be instructed to open their mouth wide, wipe the swab around their tonsils on both sides of their throat, place it in the transport tube, screw the transport tube’s cap back on securely, and finally place the tube in the provided biohazard bag.
3. Rectal swab self-collection kit (for gonorrhea and chlamydia testing): Participants will be instructed to apply 1 drop of lubricant to the tip of the swab, insert it approximately 1.5 inches into their rectum, gently rotate it for 5 to 10 seconds in a circular motion, withdraw it carefully and place it in the transport tube, screw the transport tube’s cap back on securely, and finally place the tube in the provided biohazard bag.
4. Urine specimen self-collection kit (for gonorrhea and chlamydia testing): Participants will be instructed to collect the first part of their urine stream into a sample collection cup marked with a fill line, use a pipette to transfer urine from the cup into the transport tube, repeating the process until the tube is filled between the minimum and maximum fill lines, screw the transport tube’s cap back on securely, and finally place the tube in the provided biohazard bag.
  Once all specimens (intended to be returned by a participant) have been placed in the biohazard bag, participants will be informed that they should seal it and place it in the prepaid shipping envelope #1 to be returned to the Emory University Clinical Virology Research Laboratory.
5. Hair sample self-collection kit (to assess adequacy for potential PrEP adherence monitoring in the future): Participants will be instructed to clean scissor blades with the alcohol wipe, cut a segment of hair (about 20 to 30 fibers) from the side of their head as close to their scalp as possible, tape the hair sample to the piece of aluminum foil with an adhesive label placed on the hair end furthest from the scalp, fold the foil shut, and finally place it in the clear plastic bag provided.
  Once the hair sample (intended to be returned by a participant) has been placed in the clear plastic bag, participants will be informed they should seal it and place it in the prepaid shipping envelope #2 to be returned to the HAL at UCSF.

Specimen return is completely voluntary, and no incentives will be provided to participants for completing this step. Returned finger-stick blood samples, pharyngeal swabs, rectal swabs, and urine specimens will be tested for HIV, gonorrhea, and chlamydia at the Emory University Clinical Virology Research Laboratory and returned hair samples will be visually inspected for adequacy for PrEP drug level testing at the HAL at UCSF. HAL staff have analyzed tens of thousands of hair samples for TFV and FTC concentrations and can readily determine by visual inspection if the self-collected specimens are adequate for potential PrEP adherence monitoring. No identifiable information will be provided to laboratory personnel at Emory University or UCSF, and the specimens will be connected to the results solely on the basis of the box ID. Results will be returned to the study staff at UMSN through a password-protected file shared over Box, a secure cloud storage and collaboration platform approved by the University of Michigan.

HIV, gonorrhea, and chlamydia test results will be delivered back to participants by trained counselors over the phone. Each of the counselors will have experience in the provision of HIV Counseling, Testing, and Referral. They will therefore have experience in answering participants’ questions about the HIV and STI testing processes, addressing concerns around sexual risk behaviors, and initiating linkage to care. For anyone testing positive for HIV, gonorrhea, or chlamydia, the study staff will compile a list of local treatment providers in their area using resources such as United Way 2-1-1 [105] and Emory University’s AIDSVu testing and treatment locator [106]. Participants with positive test results will be counseled about the importance of accessing treatment, notifying their sexual partners, and sexual risk reduction measures. Within 24 hours of delivering the positive test results, the study staff will send them an email including a list of local treatment providers. Participants will be contacted by phone 2 more times: (1) 2 weeks after the initial delivery of positive test results to confirm whether or not an appointment was made and (2) 4 weeks after the initial delivery of positive test results to assess engagement in care and provide any additional resources requested. Participants testing negative for HIV, gonorrhea, and chlamydia will be counseled about the importance of regular screening and engaging in safe sexual behaviors. For anyone testing negative for HIV and not on PrEP, the study staff will provide more information about this prevention option. During the phone call to return HIV and STI test results, participants will also be informed about whether the self-collected hair samples they returned were of adequate quality for potential PrEP adherence monitoring. If a participant’s hair sample quality analysis results have not been received before their HIV, gonorrhea, and chlamydia results, the study staff will follow-up with participants as soon as they are available (ie, the return of HIV and STI test results will be prioritized).

Participants will be given 6 weeks from box delivery to collect their biological specimens and return them by mail for laboratory processing. Those who have returned some or all of their specimens will be emailed a link to a short posttest survey, within 24 hours of the results delivery phone call. The survey, programmed in Qualtrics, will assess any change in their previous willingness and perceived ability to self-collect and return different types of specimens since using the specimen self-collection kits. Participants who have not returned any specimens within 6 weeks of box delivery will also be emailed a link to the posttest survey at that time point to assess any change in their previous willingness and perceived ability to self-collect and return specimens after seeing or attempting to use the 5 different types of specimen self-collection kits. The survey will also include questions to elicit reasons for not returning each type of specimen using lists of pre-specified options, as well as open-ended response fields. The posttest survey will take approximately 10 min to complete, and participants will receive an incentive of US $10 in the form of an Amazon gift card.

Similar to the baseline survey, after participants have been sent the original email containing a link to the posttest survey, they will receive up to 3 reminders at weekly intervals using their preferred communication method indicated during registration (email, phone call, or text). The final reminder will advise participants that noncompletion of the survey within the next 7 days will suggest that they are no longer interested in continuing, unless they contact study staff and ask for an extension. Participants who do not complete the posttest survey within 7 days of receiving the final reminder (ie, by the end of 4 weeks after the original email has been sent) will no longer be contacted.

### Phase 1 Outcomes

Specific outcomes to be measured during this phase include— *Information:* (1) Pre-existing awareness of commercially available self-collection kits for home-based HIV testing, (2) Knowledge of transmission and prevention of HIV and other STIs, and (3) Variations in knowledge levels across categories of selected characteristics (eg, age, race and ethnicity, educational level, sexual orientation, HIV and bacterial STI testing history, relationship status, sexual behaviors, experiences with medical care, substance use, and psychological distress); *Motivation:* (1) Theoretical willingness to self-collect and return each of the 5 different types of biological specimens, (2) Actual return of each type of specimen, defined as the receipt of prepaid shipping envelopes back at the laboratories within 6 weeks of box delivery to participants, and (3) Reasons for not returning certain types of specimens; and *Behavioral skills:* (1) Perceived ability to self-collect and return each of the 5 different types of biological specimens and (2) Adequacy of specimens to conduct actual laboratory testing for HIV, gonorrhea, and chlamydia (determined by testing returned finger-stick blood samples, pharyngeal swabs, rectal swabs, and urine specimens) and potential laboratory testing for PrEP drug levels (determined by visually inspecting returned hair samples).

### Phase 1 Analytic Plan

Descriptive statistics (means, medians, and interquartile ranges for continuous variables, and counts and proportions for categorical variables) will be used to characterize the demographic and behavioral characteristics of the sample (including HIV and bacterial STI testing history) using software for quantitative data analysis (SAS). Analyses paralleling the IMB model include— *Information:* (1) Proportions of participants who are aware of commercially available self-collection kits for home-based HIV testing will be estimated, (2) Scores for the knowledge of transmission and prevention of HIV and other STIs will be formulated for each participant, and (3) Variations in knowledge levels across categories of selected characteristics will be assessed using chi-square tests for homogeneity; *Motivation:* (1) Frequency distributions of participants’ theoretical willingness to self-collect and return each of the 5 different types of biological specimens will be plotted, (2) Proportions of each type of specimen actually returned will be calculated and compared with theoretical willingness using Fisher exact tests across categories of selected characteristics (eg, age, race and ethnicity, educational level, sexual orientation, HIV and bacterial STI testing history, relationship status, sexual behaviors, experiences with medical care, substance use, and psychological distress), and (3) Reasons for not returning certain types of specimens will be tabulated, including manually reviewing and reassigning open-ended responses to appropriate pre-specified options; and *Behavioral skills:* (1) Frequency distributions of participants’ perceived ability to self-collect and return each of the 5 different types of biological specimens will be plotted and (2) Proportions of each type of returned specimen that are deemed adequate for actual laboratory testing (for HIV, gonorrhea, and chlamydia) and potential laboratory testing (for PrEP drug levels) will be calculated. Frequencies and proportions of positive and negative HIV, gonorrhea, and chlamydia test results and linkage to care statistics will also be aggregated.

### Phase 2 Procedures

The study staff will review participant records to identify 2 subsamples of individuals including those who (1) returned all 5 specimens and (2) returned some or no specimens. Up to 16 participants from each group (32 total) will be invited to participate in individual in-depth interviews to be conducted using VSee, a video-based teleconferencing software. VSee uses Federal Information Processing Standard Publication 140-2 certified encryption and does not stream data through a third party, promoting compliance with the Health Insurance Portability and Accountability Act. Within each subsample of 16 participants, quota sampling will be used to ensure equal numbers of black and white MSM including those of Hispanic ethnicity.

Invitations will be extended via email, phone calls, or texts depending upon participants’ preferred communication methods indicated during registration. Participants will receive up to 3 reminders at weekly intervals requesting participation in an in-depth interview. The final reminder will advise participants that not contacting study staff within the next 7 days will suggest that they are no longer interested in continuing, unless they ask for an extension. Participants who do not respond within 7 days of receiving the final reminder (ie, by the end of 4 weeks after originally being requested to participate in an in-depth interview) will be thanked for their interest in the study and emailed information about the CDC HIV Risk Reduction Tool website.

Participants who agree to an in-depth interview will be given a range of dates and times to choose from and be emailed instructions to download the VSee app on their computers (to be used with a webcam) or their mobile phones (to be used with their phone’s front-facing camera). One-on-one interviews will be conducted by the study staff using a desktop computer equipped with a webcam, and each session will be audio-recorded using a digital device to allow for verbatim transcription. Audio recordings of the interviews will be deleted on an ongoing basis as soon as the transcription is complete. Each in-depth interview will take approximately 45 min to complete, and participants will receive an incentive of US $40 in the form of an Amazon gift card.

### Phase 2 Outcomes

Besides obtaining feedback on box packaging and its contents (including instructions for each specimen self-collection kit), specific domains that will be discussed in the in-depth interviews include— *Information:* (1) familiarity with the concept of biological specimen self-collection and (2) differentiating between rapid home HIV tests and specimen self-collection kits for laboratory testing; *Motivation:* (1) attitudes toward self-collecting and returning each type of biological specimen and (2) factors influencing the actual return or the failure to return certain specimens; and *Behavioral skills:* (1) ease of self-collection from different anatomical sites and (2) personal and logistical barriers (both perceived and actual) encountered.

### Phase 2 Analytic Plan

Transcribed in-depth interviews will be checked for accuracy, formatted, and imported into software for qualitative data analysis (MAXQDA). After reading some transcripts, initial codes will be created and categorized under the following theoretical constructs— *Information*: (eg, *familiarity with the concept of biological specimen self-collection*); *Motivation:* (eg, *willingness to return a finger-stick blood sample*); and *Behavioral skill* s: (eg, *ease of self-collecting a hair sample*). The coding scheme will be systematically reviewed, compared, and refined in team meetings, until we establish an intercoder reliability of ≥0.90. All transcripts will be coded using our agreed-upon coding scheme, and inductive codes will be added throughout the iterative analytic process. New themes that emerge under each construct of the IMB model will be discussed, and the codebook will be adapted as necessary. Although our qualitative analysis will be primarily led by a phenomenological approach (inductive), it will be guided by an underlying conceptual framework (deductive). Thematic analysis will be used to continue analyzing the data until theoretical saturation and redundancy across relevant domains are reached.

## Results

**Figure 2:**
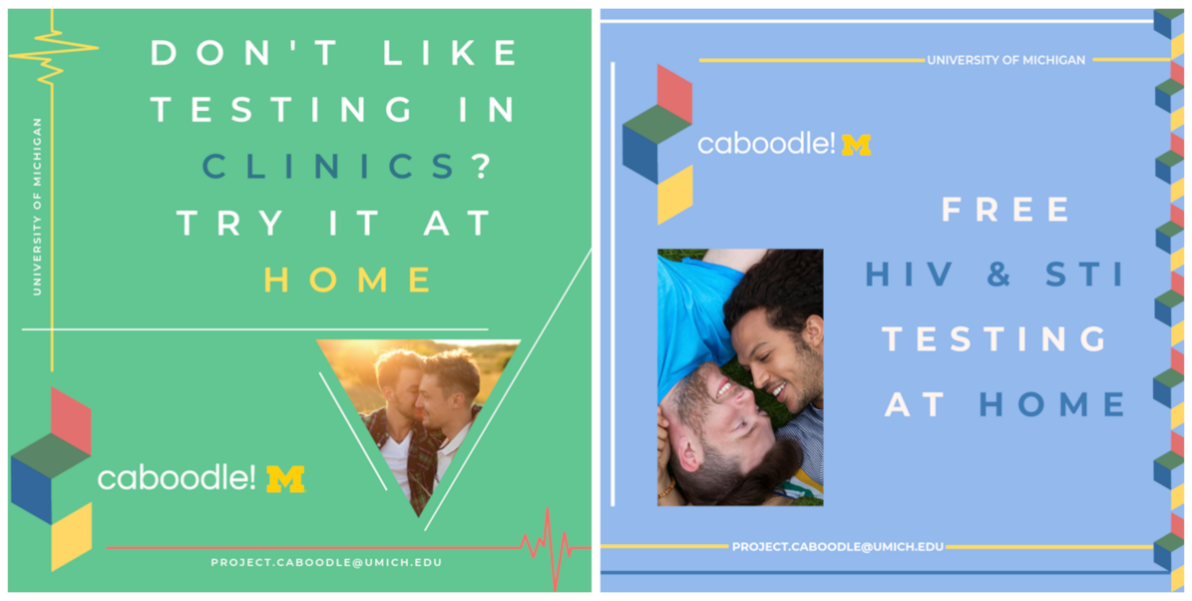
Sample advertisements for Project Caboodle!. STI: sexually transmitted infection.

Project Caboodle! was funded by the National Institutes of Health (NIH) in May 2018, and participant recruitment began in March 2019. The study team has completed the process of designing a study logo and creating advertisements for social media platforms (see Figure 2).

The Web-based informed consent document, eligibility screener, baseline survey, and posttest survey have been programmed in Qualtrics and are being tested for any inadvertent errors. The step-by-step written instructions accompanied by color images have been finalized by the laboratories that will be conducting biological specimen testing (Emory University Clinical Virology Research Laboratory and the HAL at UCSF). The boxes containing 5 specimen self-collection kits (finger-stick blood sample, pharyngeal swab, rectal swab, urine specimen, and hair sample) affixed with unique identification stickers are being assembled at UMSN in Ann Arbor. UPS shipping procedures (for mailing out boxes to participants), FedEx shipping procedures (for the return of specimens by participants using prepaid shipping envelopes), and payment procedures for completing the incentivized surveys and in-depth interviews (involving Amazon gift cards) are being finalized.

## Discussion

### Principal Findings

Project Caboodle! seeks to explore the acceptability and feasibility of self-collecting at home and returning by mail a bundle of 5 different types of biological specimens by young sexually active MSM residing in the United States. The potentially reduced time, expense, and travel associated with this strategy could facilitate a wider implementation of HIV and bacterial STI testing algorithms, as well as PrEP adherence monitoring in this high-risk group. Although some recent studies have focused on the acceptability of self-collecting and returning individual specimens for HIV or other STI testing (as opposed to a bundle) [75-87], none have incorporated PrEP adherence monitoring using hair samples. As hair is nonbiohazardous, easy-to-ship, and stable at room temperature, this specimen is particularly adaptable to self-collection. As PrEP moves from clinical trials into routine practice, these preliminary data can guide the future development of remote monitoring strategies for MSM. Our study will fill a critical gap in knowledge regarding feasibility by comparing the theoretical willingness and perceived ability to self-collect each of the 5 different types of biological specimens with the proportions of specimens actually returned and their adequacy for laboratory testing, respectively. High levels of theoretical willingness may overstate the actual rates of self-collection and return of certain specimens (eg, finger-stick blood, which is invasive to collect) because intentions do not always translate into behavior [107,108]. Obtaining this information is important, as naively assuming that positive intentions would translate into meaningful action could result in an inefficient deployment of limited resources in larger research studies and public health programs. Finally, gathering qualitative data on attitudes toward and barriers to self-collecting and returning both invasive and noninvasive specimens is critical to developing novel prevention interventions for MSM, with the ultimate goal of reducing their disproportionate burden of HIV and other STIs.

The potential challenges and limitations associated with our study do not outweigh the importance of conducting this research. Using social media platforms to recruit a convenience sample of young, high-risk MSM will reduce the generalizability of our findings. However, given the increasing use of websites and mobile apps by MSM to find sex partners [109-113], we believe this is an important starting point. We recognize practical issues with survey completion such as low or nonresponse, the potential for nondelivery of study boxes, and the reengagement of participants who returned some or no biological specimens for our qualitative phase. Black MSM’s general distrust of the research community and heightened perceptions of stigma [114] could result in differential return of specimens. We will not be able to validate the veracity of returned samples (ie, whether they belong to an enrolled participant), but we do not anticipate this to be a major issue as MSM can choose to not return some specimens if they so desire. Finally, although we are confident about our ability to collect data on acceptability and potential return, we acknowledge that assessing specimen adequacy for laboratory testing is dependent solely upon their actual return. We recognize that baldness or very short cropped hair may limit the ability of a participant to self-collect an adequate hair sample but hope to capture this information in our posttest survey that includes questions to elicit reasons for not returning each type of specimen.

### Conclusions

Despite these limitations, the self-collection of biological specimens for HIV and bacterial STI testing as well as PrEP adherence monitoring and their return by mail for laboratory testing might offer a practical and convenient solution to improve comprehensive prevention efforts for high-risk MSM. Our results will provide formative data that can be used to plan HIV and bacterial STI prevention programs and remote PrEP monitoring strategies for other minorities at risk, such as transgender men and women, as well as cisgender women [115].

## Abbreviations

CDC: Centers for Disease Control and Prevention
FTC: emtricitabine
HAL: Hair Analytical Laboratory
IMB: Information-Motivation-Behavioral skills
IP: Internet Protocol
MSM: men who have sex with men
NIH: National Institutes of Health
PrEP: pre-exposure prophylaxis
STD-KQ: Sexually Transmitted Disease Knowledge Questionnaire
STI: sexually transmitted infection
TFV: tenofovir
UCSF: University of California, San Francisco
UMSN: University of Michigan School of Nursing
UPS: United Parcel Service
